# Increase in plasma Niemann–Pick disease type C2 protein is associated with poor prognosis of sepsis

**DOI:** 10.1038/s41598-021-85478-x

**Published:** 2021-03-15

**Authors:** Yu Bai, Shuangyi Yin, Vivian Gbordzor, Yu Guo, Qing Bai, Shuaiwei Wang, Xiangyan Wei, Na Chen, Yijie Zhang, Wei Li

**Affiliations:** 1 Sepsis Laboratory, Center for Translational Medicine, Huaihe Hospital, Henan University, 115 Xi Men Blvd, Kaifeng, Henan China; 2 Department of Pulmonary and Critical Care Medicine, Huaihe Hospital, Henan University, 115 Xi Men Blvd, Kaifeng, Henan China

**Keywords:** Diseases, Medical research

## Abstract

The functional significance of extracellular Niemann–Pick disease type C2 protein (NPC2) is poorly defined. It is not known whether there is an association between plasma NPC2 and sepsis. Our exploratory, quantitative proteomic analysis showed a significant increase in the level of plasma NPC2 in moribund sepsis patients. Thus, we subsequently determined NPC2 concentration in plasma from healthy subjects, pneumonia patients and sepsis patients with comorbid pneumonia; and analyzed the association of plasma NPC2 with organ dysfunction and prognosis of sepsis patients. Our data shows that plasma NPC2 concentration was significantly higher in pneumonia and sepsis patients than healthy subjects, and was further increased in sepsis patients when the SOFA score reached 14. In addition, NPC2 concentration was significantly higher in patients that subsequently developed septic shock or died within 30 days. Moreover, NPC2 level showed the strongest association with the degree of renal dysfunction in sepsis patients. In moribund sepsis patients, however, NPC2 had highest correlation coefficient with indicators of coagulation anomaly. Based on these results, we conclude that **t**he increase in plasma NPC2 in sepsis patients is associated with multiple organ failure, possibly results from a deficiency in renal clearance, and may serve as a prognostic marker for sepsis.

## Introduction

Sepsis is considered as a life-threatening organ dysfunction caused by infection-induced host responses^1^. As a major threat to human health, sepsis is associated with as many as 11 million deaths annually worldwide^2^. Despite enormous progresses in understanding the pathophysiology of sepsis, effective treatments of sepsis are still out of reach and, as a result, organ function support remains to be the predominant treatment strategy.

According to the most recent revision, the diagnosis of sepsis entails a dysfunction assessment of 6 critical organ systems, including the heart, lung, liver, kidney, coagulation and central nervous system (CNS), manifesting a heterogeneity in impacted organs^1^. Hence, characterization of organ-specific host responses would not only advance the understanding of the underlying mechanism of sepsis pathogenesis, but also help refine treatment approaches.

Niemann–Pick disease type C2 protein (NPC2) is a small glycoprotein of 151 amino acids, and plays a critical role in the intracellular transport of unesterified cholesterol between lysosomes and other organelles^3,4^. Indeed, mutations of the *npc2* gene have been associated with storage disorders of unesterified cholesterol and sphingolipid, and are responsible for 5% of Niemann–Pick diseases, a severe neurodegenerative disorder of the CNS^5^. In addition, reduced expression of NPC2 is associated with alveolar proteinosis and macrophage accumulation in the lung, liver and spleen, in human as well as rodents, suggesting that NPC2 may play an important role in the pathophysiology of multiple tissues/organs^6,7^. However, it is not clear whether these pathological effects of NPC2-defficiency reflect a role of intracellular or extracellular NPC2.

It has been shown that NPC2 can be secreted by cultured astrocytes, lung tumor cells and LPS-activated macrophages^8–10^. In vivo, NPC2 is believed to be primarily secreted by hepatocytes, although it is also expressed in a variety of organs^11,12^. Plasma NPC2 concentration was reported to be in the range of 2–5 ng/ml in healthy human subjects, and increase to as high as 15 ng/ml under pathological conditions, such as liver cirrhosis, hepatocellular carcinoma or atherosclerotic aneurysm^12,13^. The functional significance of extracellular NPC2 is poorly understood, but may be involved in the regulation of innate immunity in multiple organs^7,14^. It is currently not known whether extracellular NPC2 is associated with organ dysfunction in sepsis.

In our exploratory investigation of sepsis mortality-associated plasma proteins, quantitative proteomic analysis revealed a significant increase in the level of plasma NPC2 in moribund sepsis patients. To fully characterize the association of plasma NPC2 with sepsis, we determined the concentration of NPC2 in the plasma from healthy individuals, patients of community-acquired pneumonia (CAP), and sepsis patients with comorbid pneumonia (SWP). In addition, we analyzed the correlation of the NPC2 concentration with values of a panel of clinical tests, the degree of organ dysfunction, occurrence of septic shock, and 30-day mortality of sepsis patients.

## Results

### Measurement of NPC2 in moderate and moribund septic plasma by DIA-MS

**Figure 1:**
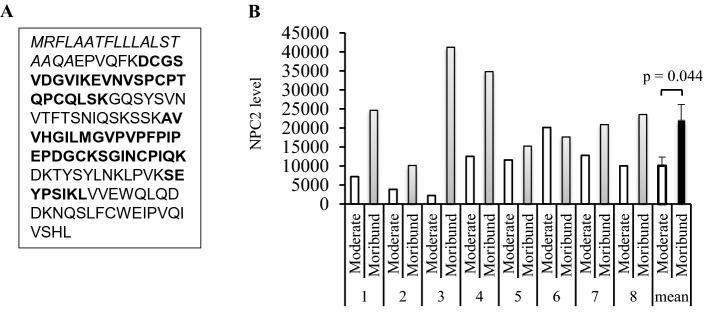
Increase in the plasma level of the Niemann–Pick disease type C2 protein (NPC2) in moribund sepsis patients. (**A**). Amino acid sequence of human NPC2. Data-dependent acquisition mass spectrometry identified 8 NPC2 unique tryptic peptides (areas in BOLD letters) in septic plasma, which overlap 49% of the NPC2 amino acid sequence, excluding the signal peptide (aa 1–19, italicized). (**B**) Data-independent mass spectrometry detected NPC2 peptides in Moderate and Moribund plasma from 8 sepsis patients. The average relative quantities of NPC2 in these plasma samples is presented in the last two columns. The p value was obtained by a two-tailed, paired t-test.

To investigate sepsis mortality-associated plasma proteins, DDA-MS was performed to construct a peptide library of septic plasma by using the 24 paired plasma samples from 12 sepsis patients. A total of 8 unique NPC2 tryptic peptides were detected, which overlapped 49% of NPC2 amino acid sequence, excluding the signal peptide (Fig. 1A). In DIA-MS analysis, NPC2 unique peptides were found in both the moderate and moribund plasma samples from 8 of the 12 patients. The level of NPC2 was higher in moribund than moderate plasma from all but one patient (#6), resulting in an average increase of 119% (*p* = 0.044, Fig. 1B). In contrast, there was no significant difference in the levels of plasma albumin between these two groups (*p* = 0.51). These results suggest that the deterioration of organ dysfunction was accompanied by an accumulation of plasma NPC2 in these sepsis patients.

### Basic characteristics of healthy, pneumonia and sepsis subjects

In light of the finding by DIA-MS, we decided to conduct a more comprehensive characterization of the association of plasma NPC2 with organ dysfunction in sepsis. Since sepsis is a subsequent development of an infection, the determination of sepsis-associated host responses entails a distinction of events that occur in sepsis from those in healthy subjects and, more importantly, in subjects with the same infection. Thus, we recruited 37 healthy individuals, and 30 CAP patients and 358 SWP patients. Further, we designated a sepsis-associated pneumonia group (SAP) including pneumonia patients that either subsequently developed into sepsis due to emergence of organ dysfunction (SOFA score ≥ 2) or evolved from sepsis after the resolution of organ dysfunction (SOFA score ≤ 1).

The basic demographic information of all cohorts, the SOFA scores and comorbidities of SAP and SWP subjects are shown in Table 1. The same day tests of mean arterial pressure and plasma lactate had been conducted in 287 SWP patients during the hospitalization, showing an occurrence of septic shock in 53 individuals, including 35 (35/196, 18.53%) males and 18 (18/90, 20%) females. Hospital records and follow-up after discharge confirmed the prognosis of 277 patients, of whom an overall 30-day mortality rate was 55.60% (154/277), including 54.10% (99/183) male and 58.51% (55/94) female patients.

**Table 1:** Demographics, organ dysfunctions and comorbidities of human subjects.

Category	Healthy	CAP	SAP	SWP
Age (years), median (interquartile)	61 (56.5–70)	69.5 (56.75–78.25)^a^	74 (66–77)^b^	71 (59–80)^c^
Gender, n (male/female)	37 (22/15)	30 (18/12)	60 (44/16)	358 (252/106)
**SOFA, mean ± SD (n)**^**d**^				
SOFA_sum	–	–	0.34 ± 0.48 (60)	7.67 ± 4.00 (358)^e^
SOFA_lung	–	–	n.d	2.70 ± 0.96 (329)
SOFA_heart	–	–	n.d	1.10 ± 1.73 (356)
SOFA_liver	–	–	n.d	0.52 ± 0.91 (330)
SOFA_kidney	–	–	n.d	0.86 ± 1.21 (351)
SOFA_coagulation	–	–	n.d	1.11 ± 1.20 (356)
SOFA_nervous system	–	–	n.d	1.67 ± 1.71 (356)
**Comorbidities, n (%)**				
Respiratory	–	30 (100)	60 (100)	358 (100)
Hypoproteinemia	–	n.d	31 (51.67)	198 (55.15)
Gastrointestinal	–	n.d	21 (35.00)	171 (47.63)
Cardiovascular	–	n.d	6 (10)	143 (39.83)
Urinary	–	n.d	4 (6.67)	93 (25.91)
Anemia	–	n.d	15 (25.00)	70 (19.50)
Neurological	–	n.d	3 (5.00)	28 (7.80)
Trauma	–	n.d	0 (0.00)	13 (3.62)
Diabetes	–	n.d	1 (1.67)	5 (1.39)
Others	–	n.d	4 (6.67)	13 (3.58)

*CAP* community-acquired pneumonia, *SAP* sepsis-associated pneumonia, *SWP* sepsis with comorbid pneumonia, *n.d.* not defined.

^a^*p* > 0.05 versus Healthy, SAP or SWP.

^b^*p* > 0.05 versus CAP or SWP, *p* < 0.01 versus Healthy.

^c^*p* > 0.05 versus CAP or SAP, *p* < 0.01 versus Healthy.

^d^SOFA scores were determined by values of tests conducted on the same day.

^e^*p* < 0.0001 versus SAP.

### Measurement of plasma NPC2 concentrations

**Figure 2:**
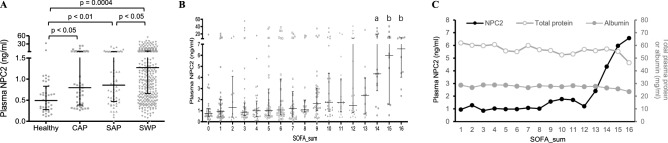
Concentration of the Niemann–Pick disease type C2 protein (NPC2) in human plasma. The concentration of plasma NPC2 was determined by ELISA. (**A**) Comparisons of plasma NPC2 concentrations in healthy subjects, and patients of community-acquired pneumonia (CAP), sepsis-associated pneumonia (SAP) and sepsis with comorbid pneumonia (SWP). (**B**) Comparisons of plasma NPC2 concentrations in pneumonia (SOFA = 0 or 1) and sepsis patients (SOFA ≥ 2) with different SOFA scores. a: *p* < 0.001 versus the group of SOFA_sum = 0; b: *p* < 0.01 versus the group of SOFA_sum = 0. (**C**) Concentrations of total protein, albumin and NPC2 in plasma from pneumonia (SOFA = 1) and sepsis patients (SOFA ≥ 2) with different SOFA scores. The Kruskal–Wallis ANOVA test followed by the Dunn’s test was used. All *p* values reflect a two-tailed testing.

NPC2 concentration in plasma samples from all groups was determined by ELISA. As shown in Fig. 2A, the median concentrations of NPC2 were 0.49 (0.28–0.83) in healthy, 0.80 (0.39–2.10) in CAP, 0.86 (0.47–1.65) in SAP and 1.28 ng/ml (0.66–3.85) in SWP groups, suggesting an increase of 63%, 76% and 161% in CAP, SAP and SWP subjects, respectively, as compared with the healthy group. The NPC2 concentration was approximately 50% higher in the SWP group than the CAP or SAP group (*p* < 0.05).

We next analyzed the concentration of plasma NPC2 in sepsis patients with different degrees of organ dysfunction. Compared with pneumonia patients (SOFA_sum = 0 and 1, Fig. 1B), no significant change in NPC2 concentration occurred in SWP patients until the SOFA score reached 14 or higher (Fig. 2B). It is important to note that there was no concomitant increase in the concentration of total protein or albumin in the plasma of SWP patients regardless of the SOFA score (Fig. 2C). Since the maximal SOFA score for each organ is 4, these results suggest that a significant increase in plasma NPC2 only occurs in patients with multiple organ failures, a condition known to be associated with poor prognosis. Consistently, among 33 SWP patients with a SOFA ≥ 14 and verified prognosis, 29 (88%) died within 30 days after sepsis diagnosis.

**Figure 3:**
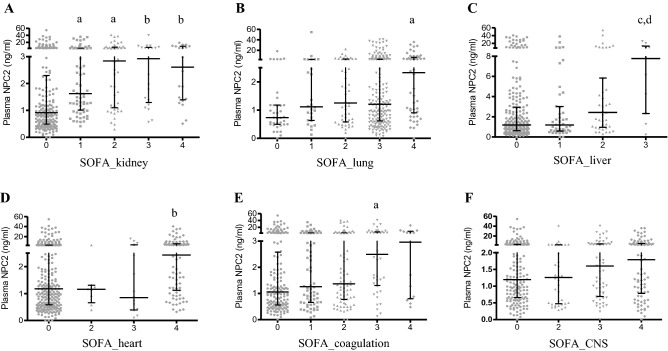
The concentrations of plasma Niemann–Pick disease type C2 protein (NPC2) in sepsis patients with dysfunction of different organ systems. The concentration of plasma NPC2 was determined by ELISA. The degrees of organ dysfunction are indicated by SOFA scores. Plasma NPC2 concentration was plotted against the SOFA scores of the kidney (**A**), lung (**B**), liver (**C**), heart (**D**), coagulation (**E**) and central nervous system (**F**). Kruskal–Wallis ANOVA test followed by the Dunn’s test was used. *p* values reflect a two-tailed testing. a: *p* < 0.01 versus the “SOFA = 0” group; b: *p* < 0.001 versus the “SOFA = 0” group; c: *p* < 0.05 versus the “SOFA = 0” group; d: *p* < 0.05 versus the “SOFA = 1” group.

No significant difference in NPC2 concentrations was found between male and female sepsis patients (Table 2). Among all comorbidity groups, sepsis patients with urinary disorders had the highest NPC2 concentration (Table 2). Interestingly, there was a significant increase in plasma NPC2 in all sepsis patients with kidney dysfunction (Fig. 3A). In contrast, an increase in NPC2 concentration became statistically significant only when severe dysfunction occurred in the lung (SOFA = 4, Fig. 3B), liver (SOFA = 4, Fig. 3C), heart (SOFA = 4, Fig. 3D) or coagulation (SOFA = 3; Fig. 3E). Given the well-characterized association between NPC2 mutations and neural degeneration in the CNS, it is somewhat surprising to note that no significant increase in plasma NPC2 was found in sepsis patients with CNS dysfunction (Fig. 3F).

**Table 2:** Plasma NPC2 concentrations in sepsis patients of different genders and comorbidities.

Comorbidity	All	n	Male	n	Female	n	Male/Female, % (*p*)
Respiratory	1.28 (0.66–3.85)	358	1.31 (0.65–3.99)	252	1.20 (0.66–3.07)	106	109% (0.58)
Hypoproteinemia	1.18 (0.67–3.29)	198	1.20 (0.66–3.45)	135	1.17 (0.80–2.60)	63	103% (0.70)
Gastrointestinal	1.36 (0.69–4.55)	171	1.38 (0.71–5.47)	115	1.20 (0.63–4.08)	55	115% (0.38)
Cardiac and vascular	1.36 (0.75–3.45)	143	1.27 (0.71–3.06)	102	1.71 (0.82–4.36)	41	74% (0.21)
Urinary	1.91 (0.85–4.33)	93	1.89 (0.69–4.00)	55	1.92 (0.96–7.58)	38	98% (0.38)
Anemia	1.18 (0.69–3.93)	70	1.22 (0.62–3.73)	52	1.08 (0.69–3.96)	18	113%, (0.88)
Neurological	1.35 (0.67–5.80)	28	1.15 (0.46–6.92)	20			
Trauma	0.98 (0.56–3.04)	13					

NPC2 concentrations are expressed in median (interquartile range, ng/ml). The Mann–Whitney U test was used to compare plasma NPC concentrations in male and female patients.

### Association of plasma NPC2 with sepsis prognosis

**Figure 4:**
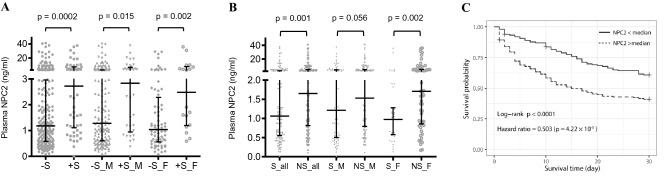
The association of plasma Niemann–Pick disease type C2 protein (NPC2) with sepsis prognosis The concentration of plasma NPC2 in sepsis patients was determined by ELISA. (**A**) Comparison (Mann–Whitney U test) of NPC2 concentrations in sepsis patients that subsequently did or did not develop septic shock. − S, no septic shock, n = 233; + S, septic shock, n = 54; − S_M: male no septic shock, n = 161; + S_M: male septic shock, n = 36; − S_F: female no septic shock, n = 72; + S_F: female septic shock, n = 18. Mann–Whitney U test was used. (**B**) Comparison (Mann–Whitney U test) of plasma NPC2 concentrations in sepsis patients that survived (S) or did not survive (NS) within 30 days after sepsis diagnosis. S_all: all survivors, n = 123; NS_all, all nonsurvivors, n = 188; S_M: male survivors, n = 84; NS_M: male nonsurvivors, n = 133; S_F: female survivors, n = 39; NS_F: female nonsurvivors, n = 55. (**C**) Sepsis patients were separated into high (> median) and low (< median) plasma NPC2 groups. The Kaplan–Meier survival curves of both groups were compared using the log-rank method. NPC2 concentration-associated hazard ratios of these two groups were estimated by Cox regression analysis. All *p* values reflect a two-tailed testing.

The NPC2 concentration was significantly higher in patients that would subsequently develop septic shock (*p* = 0.0002, Fig. 4A), in both genders. Moreover, patients that died within 30 days after diagnosis had a significantly higher concentration of NPC2 than the survivors (*p* = 0.001, Fig. 4B). Log-rank analysis of Kaplan–Meier survival curves showed that sepsis patients with a higher plasma NPC2 concentration (> median) had a higher 30-day mortality rate than those with a lower (< median) concentration (*p* < 0.0001, Fig. 4C). Consistently, Cox regression analysis showed that the hazard ratio of the low NPC2 group vs the high NPC2 groups was 0.503 (*p* = 4.22 × 10^–5^, Fig. 4C).

ROC analysis showed that AUC values of NPC2 were 0.67 (0.64 for male and 0.74 for female patients) for its association with the occurrence of septic shock, and 0.61 (0.58 for male and 0.69 for female patients) for its association with 30-day mortality. Interestingly, these AUC values were in the same range as those of common sepsis prognosis markers, such as procalcitonin and c-reactive protein, or indicators of organ dysfunction, such as platelet counts and plasma creatinine or total bilirubin (S-Table 2).

### Association of plasma NPC2 with organ dysfunction in sepsis

As shown in Table 3, correlation analysis revealed that plasma NPC2 had the strongest association with renal dysfunction (r_s_ = 0.36), followed by the overall level of organ dysfunction (SOFA_sum, r_s_ = 0.32), and dysfunctions of heart and coagulation (r_s_ = 0.2). No significant correlation was found between the concentration of NPC2 and dysfunction of the lung or CNS. Among 44 laboratory tests (S-Table 1), plasma NPC2 was most strongly associated with α-Hydroxybutyric dehydrogenase and creatinine (r_s_ = 0.37), followed by lactate dehydrogenase and other blood tests of sepsis patients (Table 3).

**Table 3:** Association of plasma NPC2 with SOFA scores and values of clinical tests of sepsis patients.

Test	r_s_	*p*	n
**SOFA**			
SOFA_kidney	0.36	10^–12^	350
SOFA_sum	0.32	10^–10^	358
SOFA_heart	0.20	10^–4^	355
SOFA_coagulation	0.20	10^–4^	355
SOFA_liver	0.17	0.002	329
SOFA_lung	0.10	0.076	329
SOFA_CNS	0.09	0.081	355
**Clinical test**			
α-Hydroxybutyric dehydrogenase	0.37	10^–9^	239
Creatinine	0.37	10^–12^	336
Lactate dehydrogenase	0.34	10^–8^	242
Blood urea nitrogen	0.29	10^–8^	338
Aspartate aminotransferase	0.29	10^–8^	337
Creatine kinase MB	0.29	10^–6^	244
Procalcitonin	0.28	10^–6^	253
Lactic acid	0.28	10^–6^	255
Fibrinogen degradation product	0.27	10^–6^	290
N-terminal pro-brain natriuretic peptide	0.27	10^–4^	157
Total bile acid	0.27	10^–6^	295
D-dimer	0.27	10^–6^	294
C-reactive protein	0.25	10^–6^	329
Alkaline phosphatase	0.24	10^–5^	311
Prothrombin time	0.24	10^–5^	308
International normalized ratio	0.24	10^–5^	308
Activated partial thromboplastin time	0.22	10^–4^	307
Red blood cell distribution width SD	0.20	10^–4^	352
Direct bilirubin	0.20	10^–4^	318
Red blood cell distribution width-CV	0.20	10^–4^	352
Alanine aminotransferase	0.18	0.001	330
Neutrophil, %	0.16	0.002	352
Thrombin time	0.16	0.006	307
Neutrophils	0.15	0.004	352
White blood cells	0.14	0.007	352
Eosinophil, %	− 0.20	10^–4^	352
Eosinophils	− 0.18	10^–4^	352
Lymphocyte, %	− 0.18	10^–4^	352
Plateletcrit	− 0.15	0.008	322

Only tests with *p* < 0.01 are listed. Correlation coefficients (r_s_) were obtained by using the Spearman’s rank-order test.

In light of the association between NPC2 level and sepsis mortality, we analyzed the correlation of plasma NPC2 and the state of organ dysfunction in moribund patients (Table 4). Coagulation parameters, such as the plateletcrit, platelet count, SOFA_coagulation and fibrinogen degradation product, exhibited highest correlation coefficients with NPC2 concentration, followed by renal dysfunction indicators, such as creatinine and urea nitrogen.

**Table 4:** Correlation of plasma NPC2 and clinical tests of moribund sepsis patients.

Test	r_s_	*p*	n
SOFA_coagulation	0.52	10^–5^	57
Fibrinogen degradation product	0.45	0.001	50
SOFA_kidney	0.44	0.001	56
Creatinine	0.43	0.001	55
Procalcitonin	0.41	0.011	39
Blood urea nitrogen	0.39	0.003	55
D-dimer	0.39	0.006	50
Mean platelet volume	0.35	0.049	33
SOFA_sum	0.31	0.015	61
Direct bilirubin	0.3	0.028	53
Total bilirubin	0.29	0.033	53
Activated partial thromboplastin time	0.29	0.039	53
Platelets	− 0.53	10^–5^	56
Plateletcrit	− 0.57	0.001	33

Correlation coefficients (r_s_) were obtained by performing the Spearman’s rank-order test.

## Discussion

Our data show a significant accumulation of NPC2 in the plasma of pneumonia and sepsis patients. A possible cause for the elevated level of plasma NPC2 could be increased synthesis and secretion. In NPC1-deficient mice, plasma NPC2 level correlates to its hepatic expression, thus leading to the suggestion that liver is a major source of circulating NPC2^11^. In human, increases in plasma NPC2 have been observed under pathological conditions, such as liver cirrhosis, atherosclerotic aortic aneurysm or hepatocellular carcinoma^12,13^, but may not be due to augmented NPC2 secretion by the liver, as hepatic expression of NPC2 was reduced in patients of liver cirrhosis^12^. Consistently, we found that the elevation of plasma NPC2 concentration in sepsis patients occurs in the absence of any increase in total plasma protein or albumin, indicators of protein synthesis activity of hepatocytes. In fact, plasma NPC2 exhibited a moderate, and positive, association with liver dysfunction in sepsis patients (i.e. increases in total bilirubin and direct bilirubin in plasma). These results suggest that the elevation in plasma NPC2 in sepsis patients may not results from an upregulation of NPC2 synthesis and secretion by the liver, but is possibly attributable to increased secretion by other tissues^12^. For instance, macrophage activation is a common inflammatory cellular response in pneumonia, sepsis as well as liver cirrhosis, and occurs in concomitant with an increase in NPC2 secretion^9^.

An alternative cause of the increased plasma NPC2 in sepsis patients could be reduced degradation or clearance. For the time being, it is not known how NPC2 is cleared from the circulation. In this study, we found that plasma NPC2 concentration was the highest in sepsis patients with comorbid urinary disorders among all comorbidities (Table 2). A significant increase in plasma NPC2 may occur when there is only a mild kidney dysfunction (Fig. 3A). In addition, plasma NPC2 exhibits a higher level of correlation with the dysfunction of kidney than other organ systems in sepsis patients (Table 3). Thus, these observations raise the possibility that renal clearance is an important mechanism for the regulation of plasma NPC2, and therefore, the occurrence of renal dysfunction would consequently lead to an accumulation of NPC2 in the circulation.

The concentration of plasma NPC2 in sepsis patients did not differ significantly from pneumonia patients until the SOFA score reached to 14, suggesting that a significant accumulation of NPC2 is associated with multiple organ failure and mortality. Consistent with this notion is that NPC2 level was significantly higher in sepsis patient when they were within 24 h from death than when they were in moderate conditions. Moreover, NPC2 concentration is predictive of the occurrence of septic shock and mortality. It is perhaps particularly noteworthy that the AUC values of NPC2 in predicting the occurrence of septic shock or sepsis mortality are at the similar level as common sepsis prognostic markers, such as procalcitonin and c-reactive protein, and dysfunction markers of kidney (i.e. plasma creatinine) and coagulation (i.e. platelet count), demonstrating the prognostic value of plasma NPC2 in sepsis.

Plasma NPC2 has been shown to regulate the cholesterol level in the blood and macrophages mobilization in multiple organs^7,14^. Given the extensively characterized impacts of lipid protein and inflammation on sepsis pathogenesis, it is reasonable to speculate that the increase in plasma NPC2 may play an active role in sepsis pathogenesis. The significant correlation between plasma NPC2 and fibrinolysis markers, such as the depletion of platelets and increase in fibrinogen degradation product, indicates an association of plasma NPC2 with hyperfibrinolysis, and perhaps internal hemorrhage and hypotension, in moribund sepsis patients.

In conclusion, this study demonstrates that plasma NPC2 undergoes an upregulation in pneumonia and a further increase in sepsis, possibly resulting from renal dysfunction. The significant increase of NPC2 in septic plasma occurs in association with multiple organ failure, and may serve as a prognostic marker for sepsis. Given the findings of this study, it is important to determine whether extracellular NPC2 is a damage-associated molecular patter, and whether the increase in plasma NPC2 contributes to sepsis pathogenesis in future studies.

## Methods

### Human subjects

Subjects of both genders between 50 and 85 years of age were qualified initially, and subsequently screened with the following exclusion criteria: pregnancy, autoimmune diseases, malignant tumor, leukemia, AIDS or surgery. All subjects were enrolled from departments in the Huaihe Hospital of Henan University, as indicated below. Prognosis was determined by hospital record or follow-up.

Thirty-seven subjects, with no existing medical conditions, were randomly enrolled from the Center of Physical Examination as healthy controls. Thirty CAP subjects were diagnosed according to a recently updated guideline^15^ and randomly enrolled from the Department of Pulmonary and Critical Care Medicine. Three hundred and fifty-eight SWP subjects fulfilled the diagnosis criteria of sepsis^1^ as well as pneumonia, and were enrolled from the Respiratory Intensive Care Unit. No significant differences were found in the gender and age between pneumonia and sepsis subjects.

### Blood collection and plasma preparation

One blood sample was obtained from each of the healthy subjects or pneumonia patients. For sepsis patients, blood samples were obtained on the day of sepsis diagnosis and at random times during patients’ hospital stay. Blood collection and plasma preparation were conducted as previously described^16^. Briefly, blood (2 ml) was drawn into standard EDTA-containing collection tubes and then centrifuged for 15 min at 2000*g*. The plasma was collected, aliquoted and stored at − 80 °C until use.

### Plasma samples for data-independent acquisition-mass spectrometry (DIA-MS) analysis

Twelve moribund patients were selected from the 358 SWP patients according the following criteria: male, 60–80 years old (71 ± 1.62, 64 to 79), diagnosed as sepsis with comorbid pneumonia, and died in hospital. Two plasma samples were selected from each patient: the first 12 samples (Moderate) were obtained when these patients were in a moderate condition, whereas the second 12 samples (Moribund) were obtained less than 24 h before death. The time interval for moderate and moribund sampling was 3.58 + 0.40 days (mean ± standard error), and the corresponding SOFA scores were 5.54 ± 0.60 and 11.58 ± 0.47 for the first and second samples (mean ± standard error, *p* = 10^–7^), respectively.

### Data-independent acquisition mass spectrometry (DIA-MS)

MS studies were conducted at Shanghai Applied Protein Technology Co., Ltd. (Shanghai, China). Methods and materials for the preparation of plasma samples, generation of tryptic peptide library of septic plasma by data-dependent acquisition mass spectrometry (DDA-MS), and peptide quantitation by DIA-MS were attached in Digital Supplement 1.

### NPC2 ELISA

Twenty-five microliter plasma from each sample was used to determine the NPC2 concentration by ELISA (SEK13341, sensitivity range: 156.25–10,000 pg/ml) from Sino Biological (Beijing, China) according to the protocol provided by the manufacturer. The concentration of NPC2 concentration in the plasma obtained on the day of sepsis diagnosis was used for subsequent analysis, unless otherwise specified.

### Statistical analysis

Variables were expressed in median and interquartile range, unless otherwise specified. The Shapiro–Wilk test showed that virtually all values analyzed in this study were in non-Gaussian distribution. Therefore, the correlation (r_s_) of NPC2 concentration with SOFA scores or other blood test values (S-Table 1) was performed using the Spearman’s rank-order correlation analysis. Group comparison was conducted using the Mann–Whitney U test for two groups, or Kruskal–Wallis ANOVA test followed by the Dunn’s test for multiple groups. Kaplan–Meier survival curves of sepsis patients were plotted for patients with the NPC2 concentration higher or lower than the median level, and the difference between survival curves was tested using the log-rank method. The hazard ratio (HR) of plasma NPC2 in sepsis patients was estimated through the Cox regression analysis. The value of area under the curve (AUC) of receiver operating characteristic (ROC) curves was used to assess the predictability of plasma NPC2 concentration, or other parameters, in the occurrence of septic shock or 30-day mortality. A two-sided *p* value of < 0.05 was set as the cut-off level for a significant difference.

### Ethical statement

The research protocol involving human subjects has been approved by the Medical Ethical Committee of the Henan University (Protocol # Protocol # 2016117) according to guidelines issued by the State Council of the People's Republic of China in 2016, and informed consent was obtained prior to a subject’s enrollment into the study.

## Supplementary Information

Supplementary Information.
