# Supplementary material for: Increase in plasma Niemann–Pick disease type C2 protein is associated with poor prognosis of sepsis
Source: Sci Rep. 2021 Mar 15;11:5907. doi: 10.1038/s41598-021-85478-x (PMC7961030; doi:10.1038/s41598-021-85478-x)
Supplement: Supplementary file 1 — Supplementary Information. [file 41598_2021_85478_MOESM1_ESM.docx]

**Increase in Plasma Niemann-Pick Disease Type C2 Protein Is Associated with Poor Prognosis of Sepsis**

Yu Bai1*, Shuangyi Yin1*, Vivian Gbordzor 1*, Yu Guo 1,2, Qing Bai1,2, Shuaiwei Wang1, Xiangyan Wei1, Na Chen2, Yijie Zhang1# & Wei Li1#

1. Sepsis Laboratory, Center for Translational Medicine, Huaihe Hospital, Henan University, 115 Xi Men Blvd, Kaifeng, Henan, China

2. Department of Pulmonary and Critical Care Medicine, Huaihe Hospital, Henan University, 115 Xi Men Blvd, Kaifeng, Henan, China

* These authors contributed equally to this study.

# Address correspondence to:

Dr. Wei Li

E-mail: 10220038@vip.henu.edu.cn

Tel: (86) 178-3925-0252

Dr. Yijie Zhang

E-mail: 13903782431@163.com

Tel: (86) 139-0378-2431

**Supplement**

**Part 1. Methods and materials for the preparation of plasma samples, generation of the tryptic peptide library by data-dependent acquisition mass spectrometry (DDA-MS) , and peptide quantitation by data-independent acquisition mass spectrometry (DIA-MS)**

*Plasma sample preparation and fractionation for data-dependent acquisition (DDA) library generation*

An aliquot (20 μl) from each of the 24 plasma samples from the male moribund SWP patients described above, was pooled for DDA library generation. To improve the dynamic range, 100 μl of pooled plasma was immunodepleted to remove the 14 most abundant human plasma proteins, using an Agilent human 14 affinity multiple removal system (5188-6559, Santa Clara, USA) coupled to a high performance liquid chromatography (LC-10AT, Shimadzu, Kyoto, Japan), according to the manufacturers’ instructions. Both of the high- abundance and low-abundance protein components were collected, and the protein concentration was determined using a Bradford Assay (5000021, BioRad, Hercules, USA) .

Protein digestion was performed according to the filter aided sample preparation procedure [Wisniewski et al., Universal sample preparation method for proteome analysis. Nat Methods. 2009 May; 6(5):359-62.]. Briefly, proteins (200 μg) were reduced in 30 μl 150 mM Tris-HCl buffer (pH 8.0) buffer containing 4% sodium dodecyl sulfate and 100 mM dithiothreitol. The detergent, dithiothreitol and other low-molecular-weight components were removed using 150 mM Tris-HCl (pH 8.0) buffer containing 8 M urea (UA buffer) by ultrafiltration (MRCF0R030, Millipore, Burlington, USA). Cysteine residues in reduced proteins were blocked in UA buffer containing 25 mM iodoacetamide. Then protein samples were washed thrice with UA buffer (100 μl) and twice with 25 mM NH_4_HCO (100 μl)_3_, and then sequentially digested in 40 μl 100 mM NH_4_HCO_3_ with 2 μg Ly-S (125-05061, Wako, Japan) for 4 hr and 2 μg trypsin (V5113, Promega, Madison, USA) for 16 hr at 37 °C, respectively. The resulting peptides were collected as a filtrate. Peptide content was estimated by UV light spectral density at 280 nm.

Post-digestion peptides were then separated into 10 fractions using Thermo Scientific™ Pierce™ High pH Reversed-Phase Peptide Fractionation Kit (84868, Thermo Scientific, Grand Island, USA). Each fraction was concentrated by vacuum centrifugation, reconstituted in 10 µl of 0.1% (v/v) formic acid, desalted on C18 Cartridges (4215SD, Sigma, St Louis, USA) and reconstituted in 40µl of 0.1% (v/v) formic acid. Then, the iRT peptides (Ki-3002-2, Biognosys, Schlieren, Switzerland) were added (sample and iRT volume ratio is 3 to 1) to correct the relative retention time differences between runs in DDA- and DIA-MS.

*DDA Mass Spectrometry Assay*

All peptide fractions were used for DDA library generation. DDA runs were injected on a Thermo Scientific Q Exactive HG/HG-X mass spectrometer (EXR0726042) connected to an Easy nLC 1200 chromatography system (LC-140, Thermo Scientific). The peptide (2 μg) was first loaded onto an EASY-Spray^TM^ C18 trap column (ES802A, Thermo scientific, Grand Island, USA), then separated on an EASY- Spray^TM^ C18 LC analytical Column (ES803, Thermo Scientific) with a linear gradient of buffer B (80% acetonitrile and 0.1% Formic acid) at a flow rate of 250 nl/min over 120 min. One full MS scan followed by 20 dd-MS scans with the following common specifications: positive ion, scan range: 300-1650 m/z. For full MS scan, the resolution was 60,000 at 200 m/z; AGC (Automatic gain control) target was 3e6; maximum IT was 25 ms and dynamic exclusion was 30.0s. For each of the dd-MS scan, the resolution was 15,000; AGC target was 5e4; maximum IT was 25ms and normalized collision energy was 27eV.

*Mass Spectrometry Assay for DIA-MS*

All 24 paired plasma samples (5μl each) were separately digested without immunodepletion and fractionated, as described above. The resultant peptides (2 μg each, with the addition of iRT peptides, as described above) were analyzed by LC-MS/MS operating in the DIA mode covering a mass range of 350– 1650m/z. Each DIA cycle contained one full MS–SIM scan (resolution: 60,000 at 200 m/z; AGC target: 3e6; maximum IT: 50ms; profile mode) and 30 DIA scans (resolution: 30,000; AGC target: 3e6; Max IT auto; normalized collision energy was 30eV). QC samples (pooled sample from equal aliquot of each sample in the experiment) were injected with DIA mode at the beginning of the MS study and after every 8 injections throughout the study, which was used to monitor the MS performance.

*Mass spectrometry data analysis*

For DDA library data, the FASTA sequence database was searched with MaxQuant software (version_1.5.3.17). The database was downloaded ( http://[www. uniprot. org](http://www.uniprot.org/)), and iRT peptide sequences were added. The search parameters were set as follows: enzyme is trypsin, max missed cleavages is 2, fixed modification is carbamidomethyl (C), dynamic modification is oxidation (M) and acetyl (Protein N-term). All reported data were based on 99% confidence for protein identification as determined by false discovery rate (FDR=N(decoy)*2/(N(decoy)+N(target)))≤ 1%. Spectral library was constructed by importing the original raw files and DDA search results into Spectronaut Pulsar X^TM^_12.0.20491.4 (Biognosys, Schlieren, Switzerland).

DIA data was analyzed with Spectronaut Pulsar X^TM^ by searching the above constructed spectral library. The main software parameters were set as follows: retention time prediction type was the dynamic iRT, interference on MS2 level correction was enabled, and cross run normalization was enabled. All results were filtered based on a Q value cutoff 0.01 (equivalent to FDR<1%).

**Part 2. S-Table 1-2.**

| S-Table 1. Clinical tests | |
| --- | --- |
| Tests (A-I) | Tests (L-W) |
| Activated partial thromboplastin time | Lactate dehydrogenase |
| Albumin | Lactic acid |
| Alkaline phosphatase | Lipase |
| Alanine aminotransferase | Lymphocyte (count and %) |
| Amylase | Mean arterial pressure |
| Aspartate aminotransferase | Monocytes (count and %) |
| Basophils (count and %) | Neutrophil (count and %) |
| Blood urea nitrogen | N-terminal pro-brain natriuretic peptide |
| C-reactive protein | Pancreatic amylase |
| Creatine kinase | Pao2/Fio2 (mm Hg) |
| Creatine kinase MB | Plateletcrit |
| Creatinine | Platelets |
| D-dimer | Procalcitonin |
| Direct bilirubin | Prothrombin activity |
| Eosinophil (count and %) | Prothrombin time |
| Fibrinogen | Red blood cell distribution width |
| Fibrinogen degradation products | Red blood cells |
| γ-glutamyltransferase | Thrombin time |
| Glasgow Coma Scale score | Total bile acid |
| Hemoglobin | Total bilirubin |
| α-hydroxybutyric dehydrogenase | Total protein |
| International normalized ratio | White blood cells |

| S-Table 2. AUC values of ROC curves of plasma NPC2 and other common sepsis-associated tests | | | | |
| --- | --- | --- | --- | --- |
| Test | AUC | n, no/yes | AUC | n, live/dead |
|  | (Septic shock) |  | (30-day mortality) |  |
| NPC2 | 0.67 | 213/48 | 0.61 | 114/169 |
| Procalcitonin | 0.64 | 163/42 | 0.53 | 79/133 |
| C-reactive protein | 0.58 | 217/47 | 0.57 | 114/170 |
| Creatinine | 0.65 | 220/50 | 0.57 | 117/174 |
| Creatine kinase | 0.7 | 152/40 | 0.52 | 80/136 |
| Total bilirubin | 0.61 | 213/48 | 0.52 | 114/169 |
| Platelets | 0.6 | 230/50 | 0.63 | 122/182 |
| Neutrophils | 0.59 | 230/50 | 0.61 | 122/182 |
| D-dimer | 0.73 | 187/48 | 0.66 | 104/152 |
